# Green Synthesis of Silver Nanoparticles Loaded Hydrogel for Wound Healing; Systematic Review

**DOI:** 10.3390/gels9070530

**Published:** 2023-06-29

**Authors:** Fahad M. Aldakheel, Marwa M. El Sayed, Dalia Mohsen, Mohammed H. Fagir, Dalia K. El Dein

**Affiliations:** 1 Department of Clinical Laboratory Sciences, College of Applied Medical Sciences, King Saud University, Riyadh 11433, Saudi Arabia; faldakheel@ksu.edu.sa; 2 Prince Sattam Chair for Epidemiology and Public Health Research, College of Medicine, King Saud University, Riyadh 11461, Saudi Arabia; 3 Chemical Engineering and Pilot Plant Department, National Research Centre, Giza 12622, Egypt; dr.marwameid@gmail.com; 4 Clinical Laboratory Sciences Program, Inaya Medical College, Riyadh 12211, Saudi Arabia; husseinfagir@inaya.edu.sa (M.H.F.); dkmohammed@inaya.edu.sa (D.K.E.D.); 5 National Research Centre, Giza 12622, Egypt

**Keywords:** wound healing, green synthesis, silver nanoparticles, characterization methods, polyacrylamide hydrogel

## Abstract

Wound healing is a biological process that involves a series of consecutive process, and its impairment can lead to chronic wounds and various complications. Recently, there has been a growing interest in employing nanotechnology to enhance wound healing. Silver nanoparticles (AgNPs) have expanded significant attention due to their wide range of applications in the medical field. The advantages of AgNPs include their easy synthesis, change their shape, and high surface area. Silver nanoparticles are very efficient for topical drug administration and wound healing because of their high ratio of surface area to volume. The efficiency of AgNPs depends on the synthesis method and the intended application. Green synthesis methods offer an eco-friendly approach by utilizing natural sources such as plant extracts and fungus. The characterization of nanoparticles plays an important character, and it is accomplished through the use of several characterization methods such as UV-Vis spectroscopy, Fourier transform infrared spectroscopy (FT-IR), scanning electron microscopy (SEM), transmission electron microscopy (TEM), and X-ray diffraction (XRD). These techniques are employed to confirm the specific characters of the prepared Silver Nanoparticles. Additionally, the review addresses the challenges and future perspectives of utilizing green-synthesized AgNPs loaded in Polyacrylamide hydrogel for wound healing applications, including the optimization of nanoparticle size, and release kinetics. Overall, this review highlights the potential of green-synthesized AgNPs loaded in Polyacrylamide hydrogel as promising for advanced wound healing therapies. There are different approaches of usage of AgNPs for wound healing such as polyacrylamide -hydrogels, and the mechanism after their antibacterial action, have been exposed.

## 1. Introduction

Wounds contain a complex and serious hazard to the health and lives of patients. Among the common issues is the presence of chronic ulcers in diabetic patients, which involve deep tissue damage. This condition is associated with the abnormal growth of fibroblasts and impaired cell migration. These factors contribute to delayed diabetic wound healing [1]. The impact of infected wounds extends beyond diabetic wounds and includes leg ulcers, arterial insufficiency, and burns. These conditions significantly affect the quality of life and lead to high morbidity and mortality rates, as well as constitute a substantial economic problem [2]. Efficient wound management is crucial due to the persistent challenge posed by wound healing in clinical settings. Extensive efforts have been dedicated to advancing wound care that focus on novel therapeutic strategies and technological advancements for the treatment of acute and chronic wounds. The complex process of wound healing involves various cell types, the extracellular matrix, and the involvement of growth factors and cytokines as signaling molecules. Although healing occurs as a continuous process, it can be divided into distinct phases: (i) coagulation and hemostasis; (ii) inflammation; (iii) proliferation; and (iv) wound remodeling accompanied by the formation of scar tissue. The appropriate approach to wound management significantly influences the clinical outcome. This review examines wound classification, the physiological aspects of wound healing, and the methodologies employed in wound management [1]. Wounds can generally be categorized as acute and chronic [3].

Due to the presence of numerous bacterial species in wounds, there is a recognized need for new antimicrobial material to combat the universal rise in antibiotic resistance. The level of resistance is escalating, predominantly against predictable antibiotics such as methicillin-resistant *Staphylococcus aureus* (MRSA) [4] and multidrug-resistant Mycobacterium tuberculosis [5]. This increasing antimicrobial resistance among pathogenic bacteria poses a significant healthcare challenge for humanity [6]. Accordingly, alternative methods are being discovered to overcome bacterial resistance, including the utilization of plant extracts [7] or a combination of antibiotics and other antibacterial materials [8], particularly nanomaterials [9]. Nanotechnology, which is a flourishing scientific field, utilizes nanoparticles with remarkable properties that are size-dependent. Nanomaterials encompass various types of nanoparticles (NPs), which can be classified as inorganic or organic nanoparticles, as well as nanocomposites, including porous materials, colloids, copolymers, and gels. Ultimately, nanoparticles and nanocomposites find applications in supports and coatings, often in the form of hydrogels.

Silver nanoparticles have been extensively studied and demonstrated to possess antimicrobial properties both in (in vitro) and in living organisms (in vivo). Their potential applications include food packaging and materials used for dressing wounds [10]. There are numerous techniques available for testing the susceptibility of bacteria to antimicrobial agents, which can be categorized into diffusion methods and dilution methods [11]. The agar disk diffusion method and antimicrobial gradient method are commonly used diffusion methods, while the broth dilution method and agar dilution method are frequently employed dilution methods [12].

Top of Form

The green synthesis of nanoparticles is a more efficient technique compared to other related methods of synthetization. Plants serve as valuable sources of various components and biochemicals that can function as stabilizing and reducing agents for the synthesis of green nanoparticles. The methods employed for green synthesis are environmentally friendly, non-toxic, cost-effective, and offer greater stability when compared to biological, physical, and chemical approaches [13]. Green-synthesized metal and metal oxide nanoparticles have gained significant applications in the biomedical field, including diagnostics, wound healing, tissue treatment, immunotherapy, regenerative medicine, dentistry, and biosensing. The production of metal nanoparticles using green synthesis involves extracting components from various parts of plants. These methods are environmentally friendly, safe, and cost-effective. In comparison to physical and chemical approaches, green-synthesized nanoparticles demonstrate higher efficiency in eliminating dyes, antibiotics, and metal ions [14]. Green synthesis is considered to be the optimal technique for nanoparticle preparation, as it reduces toxicity, enhances stability, and offers eco-friendly and cost-effective solutions. The use of green synthesis methods brings significant advantages in environmental and biomedical applications [15]. Green synthesis methods used for producing metal nanoparticles have gained recognition for their environmentally friendly nature, non-toxicity, and cost-effectiveness. Moreover, these methods have become increasingly important in the medical industry [16]. The widespread utilization of metallic nanoparticles in various fields, including biology, medicine, and pharmaceuticals, has generated a high demand for these nanoparticles. Accordingly, there is a significant need for improved nanoparticle production methods. The effectiveness of metallic nanoparticles in combating human pathogenic microbes has made them particularly appealing in biomedical applications [17]. Furthermore, the significance of this study is in providing an up-to-date, comprehensive literature review on advancements in the understanding of AgNP synthesis and characteristics, an examination of the various factors affecting silver nanoparticles synthesis, and an exploration of the various hydrogel scaffolds for delivering AgNPs.

Different methods of treating wounds using silver and silver nanoparticles (AgNPs) have been suggested. These treatments can be administered directly to the wound, either as powdered AgNPs or in solution form. Another approach involves utilizing non-compact materials such as water-soluble polymers and hydrogels for wound treatment. It is important to note that there have been reported cases of bacterial resistance to AgNPs, which poses a challenge. For example, clinical isolates of Klebsiella pneumoniae and Enterobacter cloacae from burn cases have exhibited resistance to AgNPs [18]. Therefore, it is crucial to continually develop new combinations of nanomaterials, antibiotics, polymers, methods of incorporation, and techniques for reducing silver to obtain different forms of nanoparticles.

This review paper provides an overview that is specifically focused on the green synthesis and characterization of silver nanoparticles. The applications of these nanoparticles are primarily targeted at antimicrobial treatment. Green-synthesized silver nanoparticles are characterized by their eco-friendliness, non-toxicity, cost-effectiveness, and improved stability. Additionally, these nanoparticles exhibit enhanced performance when compared to other approaches.

## 2. Results and Discussion

A total of 102 publications were initially identified, with the majority coming from the identified records. After removing duplicates, 102 articles remained for further examination. Following an initial screening, 83 papers were selected for a thorough examination of the full text. The eligibility of these papers was assessed based on predefined inclusion and exclusion criteria, which resulted in the final selection of 28 articles. Throughout the process, the focus remained on the green synthesis of silver nanoparticles and their antimicrobial activities. The selected systematic reviews specifically addressed the green biosynthesis of AgNPs using plants, as well as their antimicrobial spectrum. Some systematic reviews also explored the potential antimicrobial mechanisms of AgNPs loaded in hydrogel.

**Figure 1:**
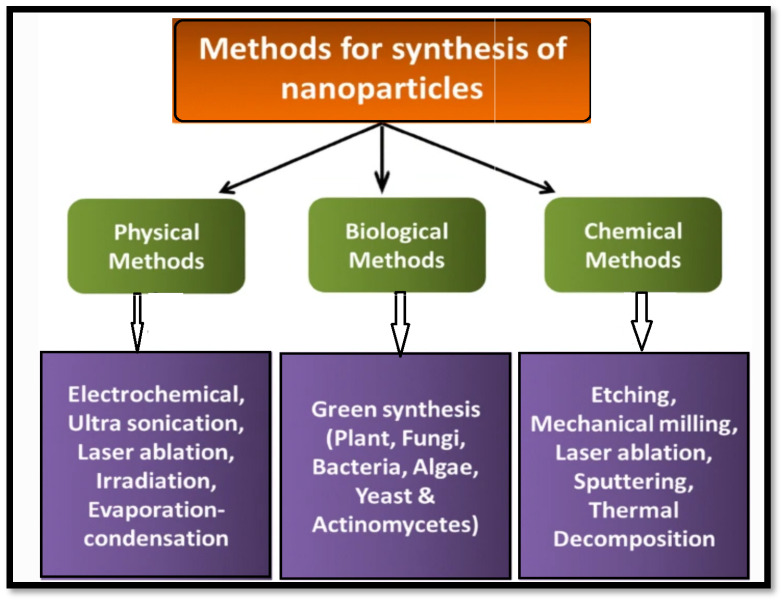
Different techniques used for the synthesis of silver nanoparticles.

Figure 1 depicts the numerous metal nanoparticle synthesis methods. In top-down synthesis, nanoparticles are produced by reducing the size of a suitable starting material to create nanoparticles. This reduction in size can be accomplished through a variety of physical and chemical processes. In top-down approaches, it is found that the product’s surface structure is imprecise, which is a significant limitation, because the surface chemistry and other physical properties of the nanoparticles are largely dependent on the surface structure.

Green synthesis discusses the process of synthesizing AgNPs by using environmentally friendly methods and reducing agents derived from natural sources. It underlines the use of sustainable and non-toxic materials in the synthesis process. Green synthesis methods characteristically involve the utilization of plant extracts, fungi, bacteria, or other biological sources as reducing agents to adapt silver ions into silver nanoparticles. These reducing agents contain bioactive compounds that assist as stabilizers and reducers, thus facilitating the formation and growth of nanoparticles. One significant advantage of green synthesis is its eco-friendliness and reduced environmental impact compared to traditional chemical synthesis methods. Green synthesis techniques often require milder reaction conditions and avoid the use of harmful chemicals, which make them more sustainable and safer for both human health and the environment. Biosynthesis, on the other hand, refers to the production of AgNPs using living organisms themselves, such as bacteria, fungi, or plants. In biosynthesis, organisms are genetically engineered or manipulated to produce and accumulate silver nanoparticles within their cells or extracellularly. The organisms act as bioreactors, where the synthesis of nanoparticles happens naturally as part of their metabolic processes.

Certain papers underwent full-text screening to expedite decision making. For example, two studies [19,20] were promptly screened for eligibility analysis. The primary objective was to identify a methodology that could resolve any disagreements and facilitate consensus.

The findings of this research indicate that green-synthesized AgNPs created through environmentally friendly methods have received support from 28 primary studies and reviews. Out of these, seven studies focused on in vitro experimentation. The green synthesis of AgNPs utilized a range of biological sources, with plants being the most common (25 studies, accounting for 89% of cases). Bacteria (2 studies, 7.1%) and fungi (1 study, 3.5%) were also used, but the majority of studies (89.28%) employed an approach with plants to green synthesize AgNPs.

### 2.1. Green Synthesis of Silver Nanoparticles

**Figure 2:**
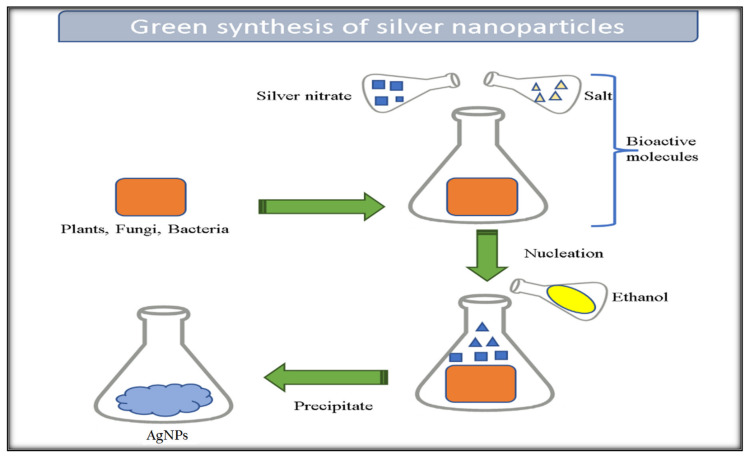
Schematic representation of the green synthesis production of silver nanoparticles (Ag NPs) using bacteria, fungus, and/or plants.

The objective of this review is to summarize the latest detections regarding the antimicrobial properties of silver nanoparticles (AgNPs) in relation to their applications in wound dressings and the process of wound healing. The nanoparticles created using a biological system possess numerous benefits, such as being non-toxic, having a high production yield, and having a clearly defined shape. As a result, it has become a novel approach to produce nanoparticles. The green synthesis method has been developed for producing highly efficient nanoparticles. These nanoparticles synthesized through green synthesis are considered safe, environmentally friendly, and simple to work with [21,22]. Stereotypically, the green synthesize method involves combining a plant, fungus, or bacteria extract with a silver-ion-containing salt, typically silver nitrate [23], where bioactive compounds present in the extracts serve to reduce the silver ions into elemental silver. One significant advantage of utilizing plant extracts is that many plants contain beneficial compounds that are known for their medicinal properties in traditional medicine and their applications in the fields of biomedicine and agriculture. Following nucleation, the AgNPs can be precipitated using an alcohol [24], such as methanol, ethanol, or isopropyl alcohol. The schematic representation of this green synthesis process is represented in Figure 1. The reduction process of Ag^+^ to Ag^0^ using biomolecules is associated with the strong reduction capacity of the mixture of biomolecules. Depending on the composition of the extract, the stirring velocity, and the time of nucleation of the AgNPs, it is possible to obtain AgNPs with varying shapes (Figure 2).

Various plant components such as roots, leaves, and seeds are employed in the production of silver nanoparticles. Previous research has indicated that silver nanoparticles can be synthesized using plant extracts [25,26]. López-Miranda and colleagues [27] highlighted the synthesis of silver nanoparticles using the plant extract from French tamarisk (Tamarix gallica). Chinnappan and colleagues also conducted a study in 2018 on this topic [28]. Duran et al. conducted a study where they utilized Fusarium oxysporum to synthesize silver nanoparticles (AgNPs) [29]. These AgNPs were then embedded into clothing materials and assessed for their ability to combat bacterial infections caused by *Staphylococcus aureus.* The results showed that the AgNPs exhibited a remarkable 99% reduction in bacterial counts [29]. In a separate study, Sundaramoorthi et al. produced silver nanoparticles (AgNPs) using AgNO3 as a precursor, and they employed Aspergillus niger as the synthesis agent [30]. These AgNPs were incorporated into wound dressings and evaluated for their antibacterial properties against various strains of bacteria. The findings revealed that the AgNPs generated inhibition zones of 15 mm against *S. aureus*, 11 mm against *Bacillus subtilis,* 10 mm against *Escherichia coli,* and 14 mm against *Pseudomonas aeruginosa* [30].

Silver nanoparticles are extensively investigated within the size range of 1 to 100 nm. They serve as an alternative method to enhance various biomedical uses, including wound healing, drug delivery, tissue scaffolding, and protective coatings. Additionally, nanosilver possesses a significant exposed surface area, thus enabling the attachment of different ligands. Silver nitrate is commonly employed for its antimicrobial properties. The field of silver nanoparticles presents a distinct and emerging approach to combat detrimental micro-organisms [31]. The physical, chemical, and biological properties of nanoparticles are highly significant. Silver nanoparticles, in particular, have numerous positive effects such as a broad-spectrum antimicrobial response, non-toxicity, anticancer properties, and other therapeutic uses. They are also capable of forming unique and diverse nanostructures and can be produced at a low cost [22].

The mechanism behind the antibacterial action of AgNPs involves several processes that contribute to their effectiveness against micro-organisms. One of the main mechanisms is the interaction between silver nanoparticles and the cell wall of bacteria. AgNPs can attach to the cell wall, thereby causing damage and disruption. This interaction interrupts the structural integrity of the cell wall, which leads to the leakage of cellular components and, ultimately, to cell death. Moreover, AgNPs can penetrate the bacterial cell membrane and enter the cytoplasm. Once inside, they can interfere with numerous cellular processes and biomolecules. Nanoparticles have been found to interact with proteins, enzymes, and genetic material (DNA and RNA) within the bacterial cell, thus leading to the inhibition of essential metabolic and enzymatic activities. This disruption hinders bacterial growth and replication. Additionally, AGNPs possess inherent properties that enable them to generate reactive oxygen species (ROS) upon interaction with bacterial cells. ROS, such as superoxide radicals and hydrogen peroxide, are highly reactive molecules that can cause oxidative stress and damage to bacterial cells. The generation of ROS by AGNPs contributes to the antibacterial effect by inducing cellular oxidative damage and further disrupting bacterial metabolism. Overall, the antibacterial action of silver nanoparticles involves a combination of physical interaction with the cell wall, the disruption of cellular processes, and the generation of reactive oxygen species. These mechanisms collectively contribute to the effective elimination of micro-organisms and their potential application in wound healing and antimicrobial treatments [32]. Researchers have found that the bactericidal properties of silver nanoparticles depend on their size and shape. Decreasing or increasing the size range of the nanoparticles influences the antibacterial response. Additionally, silver nanoparticles can assume different shapes such as spherical, rod-shaped, and truncated triangular. Among these shapes, the truncated triangular silver nanoparticles exhibit particularly strong antibacterial effects against *E. coli.* This is attributed to their high contact area and surface reactivity. The extract derived from the leaves of the black pepper plant (scientifically known as Piper nigrum) has been used to produce silver nanoparticles. These nanoparticles exhibit a specific factor that inhibits the action of certain cytokines, such as IL-6. This information is supported by reference [13]. The study observed that extracts from the European cranberry (*Viburnum opulus*) bush fruit, when used to synthesize silver nanoparticles, demonstrated anti-inflammatory effects through both in vitro and in vivo methods. These silver nanoparticles were utilized to enhance the potential therapeutic analysis for treating inflammation. Silver nanoparticles have proven to be highly effective in various biomedical applications, including antimicrobial, antifungal, antiviral, catalysis, wound healing, dressing, implanted materials, tissue engineering, anticancer therapy, and medical devices such as catheters, prostheses, and vascular grafts. Furthermore, silver nanoparticles have been utilized in certain diagnostic processes, including antipermeability factors, biosensing, and dental preparations [33].

#### 2.1.1. Bacteria-Facilitated Production of Silver Nanoparticles

Since the initial discovery by Tanja Klaus and colleagues in 1999, where they observed the clumping of silver nanoparticles (AgNPs) in *Pseudomonas stutzeri* AG259 [34], various types of bacteria, both Gram-negative and Gram-positive, have been investigated for their ability to synthesize AgNPs (Table 1). The survival capability of bacteria in environments rich in silver may contribute to the accumulation of AgNPs [34,35]. The synthesis of AgNPs can happen either within the bacterial cells or outside of them by utilizing different components such as cell-free extracts and derived substances from the bacteria. The extracellular method of synthesizing AgNPs is preferred over the intracellular method due to the ease of improving the nanoparticles. Different strains of bacteria employ distinct capabilities and mechanisms in the biosynthesis of AgNPs, which are primarily driven by organic substances. Various organic substances within bacteria can function as reducing agents, including exopolysaccharides, peptides, reductases, cofactors, and silver-resistant genes. Enzymes, such as lactate dehydrogenase, and peptides with specific amino acids, such as methionine, cysteine, lysine, and arginine, play roles in synthesizing AgNPs by attaching to the nuclei’s surface and acting as reducing agents [36]. Lactate dehydrogenase, an NADH-dependent enzyme, has gained significant attention in bacterial-mediated AgNP synthesis. It participates in the electron transport chain and creates a reducing environment by transferring hydrogen atoms. The enzyme accepts electrons from NADH, oxidizes it to NAD+, and reduces silver ions to AgNPs. Some organic substances also act as stabilizers and capping agents by preventing particle aggregation [37].

#### 2.1.2. Fungi-Facilitated Production of Silver Nanoparticles

The use of fungi for synthesizing AgNPs is a straightforward and effective approach [40]. Depending on the location of the nanoparticles, fungi-mediated synthesis can yield intracellular AgNPs using extracellular AgNPs that use a fungal cell-free filtrate [41,42] (Table 2). Compared to intracellular synthesis, the extracellular method using fungi is preferred for its ease of collection and subsequent processing. Numerous fungi species are designated for AgNP biosynthesis due to their unique abilities in metal bioconcentration, high tolerance in metal-rich environments, secretion of various extracellular enzymes, and economic viability. Examples include Fusarium oxysporum [43] and Penicillium polonicum [44]. However, it is important to note that certain fungi, such as F. oxysporum [40], are known potential pathogens, which can pose health risks in subsequent applications. The extracellular synthesis of AgNPs using fungal extracts allows for purification through washing or precipitation to remove unnecessary fungal components. Different organic components of fungi play crucial roles in AgNP synthesis, such as nitrate-dependent reductase, naphthoquinones and anthraquinones, and quinine derivatives, which are involved in silver precursor reduction. Additionally, proteins secreted by fungi can act as capping agents that influence the shape of AgNPs.

#### 2.1.3. Synthesis of Silver Nanoparticles Using Plants

In recent years, there has been significant interest in the plant-mediated synthesis of silver nanoparticles (AgNPs) as a promising approach(Table 3). This method comprises using extracts obtained from different parts of plants, such as bark, peel, callus, leaves, flowers, fruits, stems, seeds, and rhizomes, to synthesize AgNPs with varying sizes and shapes. These plant extracts contain organic components such as enzymes, alcohols, flavonoids, alkaloids, quinines, oils, terpenoids, and phenolic compounds. These organic components possess functional groups such as hydroxyl, carbonyl, and amidogen groups, which contribute to the reduction of silver ions (Ag^+^) to silver (Ag^0^). Furthermore, plant extracts and derivatives such as starch, cellulose, chitin, dextran, and alginates serve as both reducing agents and stabilizers during the synthesis process. The synthesis of AgNPs mediated by plants is influenced by various reaction parameters, including temperature, reaction time, pH, and the concentration of plant extracts and precursors. By manipulating these parameters, AgNPs with different sizes and shapes can be obtained. It is worth noting that different plant parts exhibit varying capabilities in the synthesis of AgNPs. Further examination is needed to understand the mechanisms underlying the plant-mediated synthesis of AgNPs [45].

### 2.2. Silver Nanoparticles–Polyacrylamide Hydrogel

In the study conducted by Haleem et al. [50], silver nanoparticles (AgNPs) were incorporated into a gel made of poly (N-isopropylacrylamide-co-acrylamido-2-methylpropane sulfonic acid) (NIPAMSA) to facilitate the reduction of 4-nitrophenol to 4-aminophenol. Similarly, the study conducted by [51] exhibited that chitosan-grafted polyvinyl alcohol that included AgNPs had excellent antibacterial activity against *E. coli* and *S. aureus*. The hydrogel exhibited a maximum adsorption capacity of 158.21 mg/g and 182.53 mg/g for Hg^2+^ ions at a pH of seven and six, respectively, in aqueous solutions [52]. Likewise, Dil [53] and Sadeghi 2018 [52] developed a hydrogel composed of nanosilver, gelatin, and poly (acrylic acid) (PAA) for the removal of Cu^2+^ ions. The hydrogel demonstrated an absorption capacity of 147.10 mg/g at a pH of 5.5 for a duration of 40 min, as measured by atomic absorption spectroscopy [52,53]. AgNPs–hydrogels were applied for wound treatment, as illustrated in Table 4.

The incorporation process involved loading in-situ-prepared silver nanoparticles (AgNPs) into a hydrogel composed of Carbopol. Chitosan was utilized as a capping and reducing agent. The optimized hydrogel exhibited enhanced antimicrobial activity and wound healing properties, as evidenced by its superior bactericidal effects and accelerated wound closure, which were confirmed by histopathological findings [54].

Chitosan polyacrylamide hydrogels were synthesized and exhibited significant efficacy against *S. aureus* and *E. coli* with a minimum of 99.7% inhibition of bacterial growth. Furthermore, hydrogels containing silver nanoparticles did not have any harmful effects on the skin cells that were directly cultured on them [55], as illustrated in Table 5.

### 2.3. Factors Affecting the Green Synthesis of Silver Nanoparticles

The characterization of the prepared silver nanoparticle morphology in green synthesis can be modified based on various specifications, including temperature, pH, and reaction time. These factors have been extensively acknowledged as important in the production of nanoparticles, and they may also play a crucial role in optimizing the synthesis of nanoparticles [59].

#### 2.3.1. Temperature

Globally, scientists are conducting various research studies to understand how temperature affects prepared nanoparticles. Temperature greatly impacts the size, shape, and synthesis of nanoparticles. By adjusting the temperature, scientists can customize the shape and size of nanoparticles, such as triangles, octahedrals, spheres, and rods. Higher temperatures also lead to increased reaction rates and the formation of nucleation centers. Additionally, during the green synthesis of nanoparticles, the reaction time plays a crucial role in determining the shape, size, and yield of the synthesized nanoparticles [60].

#### 2.3.2. The pH Value

The pH of a solution is vital for the structure and formation of nanoparticles. It affects the chemical setting and ionization of molecules, which influences how nanoparticles nucleate and grow. When the pH increases, the solution becomes more basic. This creates more nucleation centers, which are where nanoparticles begin to form. Higher pH levels result in more nucleation centers, thus promoting the formation of metal nanoparticles. Moreover, pH influences the shape and size of nanoparticles. Different pH conditions lead to variations in the arrangement and morphology of the nanoparticles. The pH affects reaction rates and intermediate stability during the formation process [38,61].

#### 2.3.3. Reaction Time

The crucial factor governing the structure and shape of nanoparticles is the speed at which the reaction occurs, in addition to temperature and pH. According to [62], the duration of the reaction has a significant impact on the creation of magnetic nanoparticles [63].

### 2.4. Silver Nanomaterials Characterization

**Figure 3:**
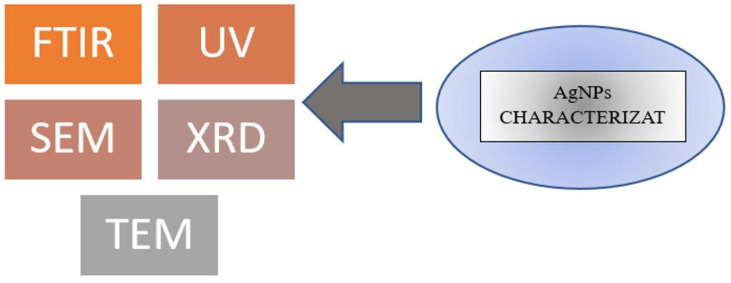
The summary of the characterization methods used for silver nanoparticles.

The classification of nanoparticle characterization can be determined based on the type of physical and chemical analysis instruments used. These instruments include UV-Vis spectroscopy, (FT-IR) Fourier transform infrared spectroscopy, scanning electron microscopes (SEMs), and X-ray diffraction (XRD) [64], as shown in Figure 3.

#### 2.4.1. UV–Vis Spectrophotometry

UV-Vis absorption spectroscopy offers an advantage in nanoparticle characterization due to its capability to detect variations in silver nanoparticle size across different metals, with a size range typically ranging from 2 to 100 nm. Commonly, the analysis of nanoparticles confirmed using UV-Vis absorption spectroscopy comprises studying wavelengths within the range of 300 to 800 nm. Silver nanoparticles synthesized under specific salt conditions exhibit strong absorption that results in a distinctive spectrum within the visible region. Previous studies have demonstrated that the absorption of wavelengths ranging from 200 to 800 nm is suitable for categorizing nanoparticle sizes within the range of 2 to 100 nm [65].

#### 2.4.2. (FT-IR) Fourier Transform Infrared Spectroscopy

In Fourier transform infrared (FT-IR) analysis, the sample is exposed to infrared rays. Some of these rays are absorbed by the sample, while the rest pass through it. By examining the spectrum, which shows the absorption or transmission of the rays at different wavelengths, one can identify the substances present in the sample [66]. FTIR analysis is a real, affordable, straightforward, and non-intrusive method to understand the role of biomolecules in reducing nanoparticles (specifically, silver nitrate to silver) [67].

#### 2.4.3. Scanning Electron Microscopy (SEM)

Scanning electron microscopy (SEM) is a technique employed to analyze silver nanoparticles, and it allows the determination of their shape, size, morphology, and distribution [68]. By utilizing SEM analysis, changes in the structural characteristics of nanoparticles can be examined before and after treatment. Prior research has indicated that significant alterations in cell shape and the presence of perforations in the cell wall serve as indicators of the antimicrobial effects of nanoparticles [69]

#### 2.4.4. Transmission Electron Microscopy (TEM)

Transmission electron microscopy (TEM) was used to classify and confirm the crystal structure and particle dimensions of a material on a nanoscale level [64].

#### 2.4.5. X-ray Diffraction (XRD)

X-ray diffraction (XRD) is a technique commonly used to analyze the atomic structures of materials. This method is valuable for determining both the qualitative and quantitative aspects of materials. In the context of nanomaterials, XRD investigations were employed to identify and authorize the size and structure of crystalline materials [64]. To evaluate the particle dimensions of nanomaterials using XRD data, the Debye–Scherrer formula was utilized. This formula involves measuring the width of the Bragg reflection law and applying the equation: d = Kλ/β cosθ. In this equation, d represents the particle size in nanometers, K is the Scherrer constant, λ denotes the X-ray wavelength, β is the full width at half maximum, and θ represents the diffraction angle, which corresponds to half of the Bragg angle and relates to the lattice plane [70].

### 2.5. Silver Nanoparticles Hydrogel for Wound Healing

#### 2.5.1. Silver Nanoparticles for Wound Healing

Experimental studies verifying the safety and efficacy of silver nanoparticle dressings swiftly led to a succession of clinical trials [71,72] that yielded on encouraging results. These studies yielded valuable information that enabled the commercialization of numerous dressings. Currently, they are used to treat venous ulcers, diabetic foot sores, burns, and surgical lesions [73].

However, several years after the first AgNP dressing was introduced to the market, the potential of silver nanoparticles in wound healing had not been completely exploited, which necessitated additional research on AgNP dressings. It is necessary to increase the analgesic and anti-inflammatory properties of the dressings, because silver does not possess them in sufficient quantities. Therefore, various varieties of biomaterials derived from natural sources were enriched with silver in order to discover the optimal dressing with all the required properties.

In 2017, Meekul et al. [74] evaluated the efficacy of an alginate silver dressing on patients with necrotizing fasciitis lesions in a clinical trial. The study examined four outcomes: wound bed preparation, hospital stay duration, pain experienced, and total hospital stay cost. The average preparation time for the wound bed was 10 days shorter in the Ag-dressing group compared to the standard treatment group (21.39 vs. 31.87 days). In addition, the length of hospitalization was shortened (20.99 versus 29.19 days). Importantly, the pain score of patients treated with alginate silver dressing was substantially lower than that of the control group, which is difficult to achieve with commercially available silver-based dressings [74].

Hahn et al. [75] investigated the effect of silver-impregnated negative pressure dressings on the number of micro-organisms in patients’ lower extremity open lesions. Participants were divided into two groups, with the control group receiving conventional negative pressure wound therapy (NPWT + polyurethane foam) and the research group receiving the same treatment, but with silver-coated polyurethane foam. Bacterial growth was reduced in the silver-treated group, and the difference between the groups widened over time. This conclusion was validated using both wound surface micro-organisms and tissue culture [75].

The team of JiHui Chen conducted an experiment in 2021 to determine the efficacy of silver-enriched dressings for treating lesions in patients with pemphigus vulgaris (PV) [76]. A total of 28 patients in the study group were treated with a dressing containing silver, whereas 30 patients in the control group were treated with gauze immersed in 0.5% povidone iodine solution. In the Ag group, the average duration of wound recovery was substantially shorter (43.72 vs. 55.00 days). Additionally, the length of hospitalization was abridged (33.72 vs. 43.64 days), and the average number of garment changes was reduced (from 43.5 to 18). Although the incidence of infection did not reach statistical significance, it is noteworthy that it was lower in the research group (1 vs. 3, *p* = 0.302). It is crucial because infection and sepsis are the leading causes of mortality among patients with PV [76]

Matilda Karlsson et al. [77] decided to conduct a prospective study comparing two treatments for partial thickness burns in children. A total of 58 children were separated into two groups, with one group receiving porcine xenografts and the other receiving a silver-foam dressing. The primary outcome was the healing time, while secondary outcomes included pain, the need for surgery, wound infection, hospitalization time, and dressing change frequency. The healing period of wounds covered with silver-based dressings was substantially shorter (15 days vs. 20.5 days). This distinction was observed in all lesions, including those that covered more than 20% of the total body surface. All other outcomes were comparable between groups, with the exception of the number and duration of dressing changes, which again favored the group treated with silver foam.

In 2022, Akin et al. [78] published the results of their study testing the efficacy of a silver dressing in reducing the risk of surgical site infection in patients following ostomy closure. It is well-known that the gastrointestinal tract is frequently colonized by multidrug-resistant bacterial strains, which makes it difficult to treat surgical site infections after colorectal surgeries. A total of 15 of the 31 participants in this study were treated with conventional gauze dressings for one to two days after the ostomy was closed. After cleansing the lesion with 10% povidone iodine, gauze dressings were applied and changed every day for five days. The lesions of the patients in the study group were treated with a silver-based dressing. The dressing was administered in the operating room and remained in place for five days following the procedure. During the 30-day period, it was observed that 26.7% of patients in the control group developed a surgical site infection, whereas no patient in the study group did. Asgari and his colleagues investigated the efficacy of silver nanoparticle dressings in the treatment of pressure ulcers in patients with spinal cord injuries in order to determine their efficacy. Seventy patients with spinal cord injuries were enlisted in the study and divided into two equal groups, one receiving a hydrocolloid bandage and the other receiving an AgNP-based dressing. The Bates–Jensen Wound Assessment Tool was used four times to evaluate the efficacy of the dressings. It was observed that ulcers resolved effectively as a result of both treatments. The relative reduction of the BWAT score for silver nanoparticles was greater, but the score was not statistically significant [78].

#### 2.5.2. Ensuring the Well-Being of Silver Nanoparticles in the Context of Wound Healing

The issue of AgNPs’ possible toxicity has been widely discussed due to their capability to easily penetrate cell membranes and potentially harm human health [79]. Studies on mice have shown that AgNPs can cross the blood–brain and blood–testis barriers [80]. Higher concentrations of silver nanoparticles (AgNPs) (>44.0 µg/mL) have been found to induce cell necrosis and rupture cell membranes (Gopinath, P.; et al., 2008). When AgNPs pass through the blood–testis barrier, they can gather in the testes and have adverse effects on sperm cells [81]. AgNPs have also been found to impair other cell types, such as brain cells [82], liver cells [83], and stem cells [84]. However, many researchers have reported that the use of AgNPs as a topical antibacterial agent in wound healing is safe. AgNP-loaded polyacrylamide hydrogels, in particular, are considered excellent wound dressings [85]. When used in appropriate quantities, silver metal and silver dressings have no negative effects on human health [86,87]. For example, a study by [88] investigated the permeability of AgNPs in human skin and their cytotoxicity in human keratinocytes under ultraviolet B (UVB) irradiation was conducted. The results displayed that AgNPs did not penetrate intact human skin and had no detrimental properties on keratinocytes or increased cell death induced by UVB. AgNPs were effective at low concentrations, did not easily penetrate the skin barrier, and had no harmful effects on keratinocytes [88]. Therefore, AgNPs have great potential for use in wound dressing applications. Additionally, nanoparticles (NPs) in general are considered a promising alternative to antibiotics due to their bactericidal activity against a wide range of pathogens, without inducing microbial resistance [89].

AgNPs, in particular, have garnered significant attention for their inhibitory effects on approximately 650 microbial species and antibiotic-resistant bacteria [90]. The antimicrobial properties of silver nanoparticles (AgNPs) have been extensively researched. AgNPs can attach to and penetrate bacterial membranes, thus leading to the subsequent destruction of the cell membrane and the leakage of cellular contents. Additionally, AgNPs can interfere with vital intracellular processes, such as disrupting the respiratory chain, hindering DNA replication, and inhibiting cell division. Moreover, AgNPs exhibit significant antimicrobial effects against drug-resistant fungi by targeting cellular components that are involved in drug resistance and pathogenicity.

Top of Form

AgNPs hinder bacterial reproduction by denaturing bacterial DNA, which leads to the alteration and subsequent death of bacterial cells [91]. Moreover, AgNPs can be operative therapeutic or prophylactic agents, as they prevent the colonization of wounds by antibiotic-resistant bacteria and other microbes that impede wound healing [92]. Studies using human keratinocytes and dermal fibroblasts have revealed that AgNPs significantly reduce levels of inflammatory cytokines and promote healing [93]. In vitro cell culture tests have confirmed no cytotoxicity in hydrogels containing AgNPs, and these hydrogels have shown non-adherent characteristics regarding dermal fibroblasts [94]. When associated with conventional topical agents, AgNPs can effectively prevent wound infections and enhance the healing process of injured tissues. AgNP-coated wound dressings exhibit strong antibacterial activity and promote faster and more efficient tissue repair [95]. The use of silver-modified nanoporous silica carriers loaded with sulfadiazine in place of silver sulfadiazine can overcome the poor aqueous solubility of the latter, which limits its antibacterial effect [96]. On the opposing side, some authors are concerned about the risks associated with AgNP usage. Several cases of argyria, a condition characterized by silver deposition, have been reported after treating burn wounds with dressings containing nanocrystalline silver [97,98]. The use of these dressings led to the deposition of silver particles in the mid and deep dermis. Silver itself has been found to have concentration-dependent cytotoxic properties in human dermal fibroblast cells [99]. Nevertheless, advancements in nanotechnology have reduced the minimum repressive concentration of silver and its toxicity to normal human cells [100], thus resulting in several brands of silver-containing wound dressings being approved by the U.S. Food authority.

### 2.6. Hydrogel

Due to the weak surface-binding affinity of AgNPs, hydrogel scaffolds have been proposed as an efficient vehicle for drug delivery. AgNPs are increasingly used in biomedicine for their antibacterial activity. In order for hydrogels to promote wound healing, they must maintain a balance between the exchange of hydration, oxygen, and chemicals. The cross-linked three-dimensional structure and hydrophilic polymer network of hydrogels allow them to function as water-retaining scaffolds, which have attracted interest in the medical field. The effectiveness of hydrogels in drug delivery, tissue engineering, and as antimicrobial agents has been demonstrated. Although a significant amount of research has been conducted on the efficacy of different hydrogel matrices, not enough has been done to compare the different varieties of hydrogel scaffolds in order to propose the most effective and stable environment for AgNP delivery [101,102].

#### Types of Hydrogel Used in Wound Healing

Polymers with zwitterionic structures that are used for AgNPs have garnered attention for their catalytic activities; however, aggregation, as with other metal nanoparticles, can drastically reduce their catalytic properties by altering their surface-to-volume ratio. The ability of zwitterionic polymers to bind water molecules and effectively reduce nonspecific protein adsorption confers antifouling properties on zwitterionic environments. This imparts hydrogel contamination resistance, which preserves the scaffold’s catalytic activity in protein-rich environments [103]. To resolve the immobilization challenges posed by AgNPs, the efficacy of zwitterionic hydrogels for preventing aggregation and increasing reusability has been investigated using polycarboxybetaine (PCB) as the zwitterionic hydrogel containing AgNPs.

The green method of 4-nitrophenol (4-NP) catalytic reduction to 4-aminophenol (4-AP) was used to observe the catalytic properties of zwitterionic hydrogels. After the homogenous distribution of AgNPs in a PCB hydrogel, the PCB–AgNPs exhibited the highest catalytic activity and reusability. This characteristic enables separation and recycling of the hydrogel via cyclic catalysis pathways. FITC-BSA was used to observe protein absorption by various hydrogel varieties. The fluorescence microscopy images obtained from the protein absorption test demonstrated results that were comparable to those of previous studies: no significant FITC-BSA attachment was observed on the surfaces of PCB, PSB, PCB–AgNP, or PSB–AgNP hydrogels [104,105]. This indicated that the catalytic activity of AgNP was not affected by the biofouling environment.

The equilibrium water content (EWC) of zwitterionic hydrogels was determined by measuring the UV-Vis absorption of the hydrogels. The high water content of hydrogels facilitates effective mass transfer. PCB–AgNP hydrogel had the maximum EWC among the AgNP hydrogels. Comparing the physical properties of AgNP-embedded hydrogels revealed that, not only did the addition of AgNPs to hydrogels not affect the compressive modulus or break strain, but the PCB–AgNP exhibited the highest physical strength [106], thus indicating a more stable matrix and greater reusability. In addition, the larger pore size of the PCB hydrogel may have enhanced its catalytic activity by facilitating greater mass transfer. The UV-Vis spectra also revealed no significant change in the AgNP concentration, thus indicating that there was no material leakage or aggregation. The research on the antibiofouling properties of PCB–AgNP has demonstrated the material’s sustained catalytic activity and recyclability, which indicates the possibility of utilizing zwitterionic hydrogels in wound-healing applications.

Nanofibrous membranes made of chitosan were created in in vivo research to evaluate the antibacterial and wound-healing efficacy of nanofibrous membranes derived from chitosan [107]. By examining the in vivo properties of AgNPs while evaluating their direct antibacterial properties, the researchers sought to fill in the voids left by previously established AgNP studies. The research was divided into in vitro and in vivo examinations of antibacterial properties, and it also involved in vivo examinations of wound-healing efficacy. Similar to the findings of a previous study, the results demonstrated that, while inorganic ions permitted the slow release of silver, proteins precluded silver release by forming a barrier [101]. Prior research investigated the effects of proteins and inorganic ions on the effectiveness of AgNP. In order to examine the effects of inorganic ions and proteins in the environment, a phosphate-buffered saline environment and fetal bovine serum (FBS) were prepared. AgNPs were immersed in both environments, and it was determined that the binding of inorganic ions to silver particles had no effect on their antibacterial properties.

In contrast, despite the fact that the FBS-immersed AgNP hydrogel exhibited a greater release rate, its antibacterial properties were not maintained in subsequent tests. The formation of a protein barrier in the FBS environment altered the hydrogel’s ability to release AgNPs. The results demonstrated the significance of investigating protein presence when determining in vitro hydrogel performance. Overall, chitosan-based nanofibrous hydrogels exhibited antibacterial properties and did not interfere with the wound-healing process. The antimicrobial properties of lignin-based hydrogels have been examined [102,108]. Previous research demonstrated that lignin, a natural lignocellulosic polymeric matrix, possesses antioxidant and antibacterial properties [103].

## 3. Conclusions

Nanomaterials have emerged as a highly promising approach for eradicating bacterial, fungal, and viral infections. In addition to their antibiotic, antifungal, and antiviral effects, they find utility as catalysts, bioremediation agents, and pseudoenzymes. This review showcases the successful outcomes achieved in the field of nanotechnology. Acute and chronic wounds pose significant challenges in terms of healing and often receive inadequate attention. Green synthesis offers a more environmentally friendly and user-friendly alternative by utilizing the natural sources of reduction agents. It enables the production of a diverse range of NPs. It is important to note that optimizing the production of uniformly synthesized green NPs can be a time-consuming process, as even slight and inadvertent changes in laboratory protocols can result in NPs with entirely distinct chemical and physical properties. Furthermore, a thorough characterization of plant or fungus extracts is necessary in green synthesis. Despite the multitude of publications in recent years, there remains a need to explore new polymers and synthesis techniques, not only for silver nanoparticles (AgNPs), but also for other types of NPs, due to the escalating bacterial resistance to metal NPs. The green synthesis method refers to an environmentally friendly, non-toxic, and cost-effective approach. In this review, we provided a summary of the green synthesis method, as well as its characterization techniques. These nanoparticles have been utilized for analyzing properties such as antibacterial, antifungal, and antioxidant properties. The findings of these studies strongly support the use of the green synthesis approach for the development of silver nanoparticles, which can have more beneficial effects in environmental and biomedical applications. In our future research, our group will focus on synthesizing different types of green nanoparticles to develop applications in various sectors, including pharmaceutical, medicine, environment, aquaculture, and agriculture sectors. The results of this study shed light on the direction of future research in the development of green nanoparticles for environmental and biomedical purposes.

## 4. Materials and Methodology

**Figure 4:**
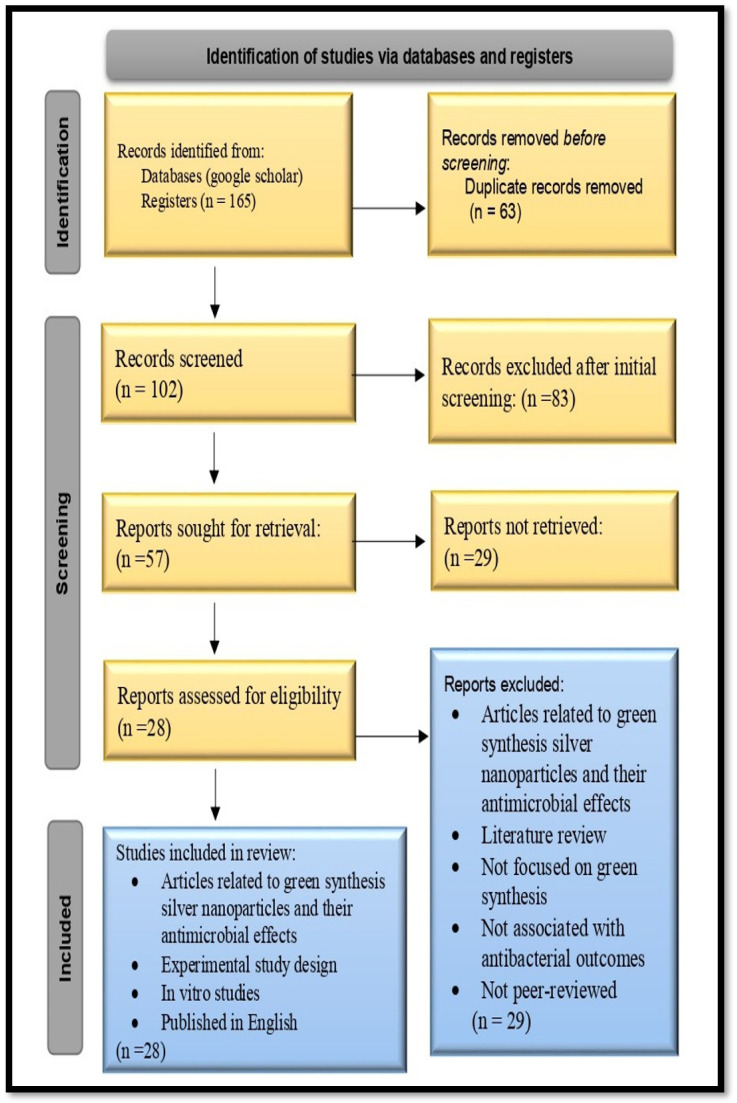
PRISMA (Preferred Reporting Items for Systematic Reviews and Meta-Analysis).

Numerous systemic reviews investigating the process of producing silver nanoparticles loaded in hydrogel with antimicrobial properties were conducted and gathered. Varieties of reports addressing this topic were discovered, and it was crucial to conduct a comprehensive review to combine the findings from these reports and reach a definite understanding while avoiding any inconsistent information or misunderstandings. This overview, with the specific goal of emphasizing the antibacterial effectiveness of biosilver nanoparticles (AgNPs) loaded in polyacrylamide hydrogel, exclusively focused on compiling all relevant systematic reviews, following the recommendations of Cooper and Koenka [20]. To document this summary, the PRISMA (Preferred Reporting Items for Systematic Reviews and Meta-Analysis) method was used (Figure 4).

### 4.1. Problem Formulation

The initial stage involved defining the issue concerning the evaluation and the establishment of AgNPs loaded in hydrogel during the investigation of their antimicrobial properties. This research question was then expanded to explore the potential of AgNP–polyacrylamide hydrogels as smart tools against many drug-resistant microorganisms, with the aim to substitute antibiotics. Furthermore, the possibility of utilizing AgNPs–polyacrylamide hydrogel as a viable treatment option and as a real antimicrobial agent was also examined.

### 4.2. Conducting Literature Searches to Gather Information for Research Syntheses

The second step involved conducting a systematic literature review in May 2023. Relevant literature was searched in three electronic databases: MEDLINE, ScienceDirect, and Scopus. Specific terms were used and taken from the title, abstract, and keywords, including terms such as “Green Synthesis of Silver Nanoparticles”, “silver nanoparticles-in Hydrogel”, “biosynthesis”, “green synthesis”, “antimicrobial resistance”, and “systematic review”. The search criteria were modified accordingly for each database, and operational definitions of keywords were obtained from selected articles. The analysis included articles containing factual information, summaries, and reviewers’ comments. Only English language articles were included in the literature search. Inclusion criteria for both phases of the search process were defined as follows: (a) articles related to green synthesis silver nanoparticles and their antimicrobial effects, (b) experimental study design, (c) in vitro studies, and (d) published in English. Exclusion criteria involved (1) reports that did not conduct a systematic search (literature review), (2) articles that were not peer-reviewed, (3) articles not focused on green synthesis, (4) articles not associated with antibacterial outcomes, and (5) articles not published in English. Duplicate papers were identified and removed prior to the selection process. Duplicate papers were defined as papers published in two journals with the same title, same first author, same study design, same sample size, and the same number of in-text citations or references. The principal author’s name was used for reviewing each retrieved research paper for inclusion or exclusion, and full texts were retrieved for all such papers until a decision was made. The included research papers were searched again to identify additional relevant articles, which then went through the same screening process to ensure the credibility and reliability of the research.

### 4.3. Extraction of the Data

A matrix table was utilized to present the selected papers, which allowed for an organized exhibition of information. A descriptive analysis was conducted to examine each article individually. Following the descriptive analysis, a comparison was made between the articles that followed the recommendations of Cooper and Koenka [20]. Relevant details such as the authors’ names, study objectives, the number of studies with feedback, identified criteria, and the results and feedback were extracted from the literature reviews. During the analysis, data were collected to verify and validate the findings and outcomes. This analysis involved identifying and describing the issues associated with each keyword.

### 4.4. Meta-Analysis Processing

The selected articles were categorized into specific domains related to AgNPs loaded in hydrogel, which included their sizes, shapes, applications, and compositions. Articles focusing on the synthesis and functions of green synthesized silver nanoparticles were grouped together. The selection process prioritized articles that specifically addressed the green synthesis of AgNPs and demonstrated effective antibacterial properties. Several steps were taken to analyze the antibacterial properties of the synthesized AgNPs. Initially, an electronic literature search was conducted to identify original research on silver nanoparticles. The selected articles were chosen based on their relevance, authenticity, recent publication, and focus on AgNP green biosynthesis with hydrogel. The corresponding or first author of the initial article was included, following the recommendation of Costas and Bordons [19], as they have been typically responsible for the report or the development of the idea. After completing five steps, the review involved integrating the results of the reviewed articles, particularly to identify any conflicting findings. A second-order meta-analysis would have been conducted if there were discordant results; however, no such discrepancies were found in the current study, thus rendering a meta-analysis unnecessary.

## Figures and Tables

**Table 1:** Synthesis of silver nanoparticles using bacteria.

Bacteria	Precursor	Functional Groups/Organic Components	Condition	Size by (nm)	References
**Pseudomonas**	AgNO_3_	Amines (Aromatic and Aliphatic)	shaking 28 °C	10–40	[38]
**Streptomyces violaceus**	AgNO_3_	Exopolysaccharide	shaking; 37 °C pH 7.0	10–60	[39]

**Table 2:** Synthesis of silver nanoparticles using fungi.

Fungi	Precursor	Functional Groups/Organic Components	Condition	Size by (nm)	References
**Penicillium polonicum**	AgNO_3_	Proteins	Shaking Room temp.light	10–15	[44]
**Fusarium oxysporum**	AgNO_3_	Proteins	shaking 28 °C	21.3–37.3	[40]

**Table 3:** Synthesis of silver nanoparticles using plants.

Bacteria	Precursor	Functional Groups/Organic Components	Condition	Size by (nm)	References
*Aloe vera* (leaf)	AgNO_3_	Hemicellulose, lignin, and pectin	100 °C or 200 °C; shaking	70.70 ± 22, 192.02 ± 53	[46]
*Ocimum sanctum* (leaf)	AgNO_3_	Quercetin	–	250–600	[47]
*Gossypium hirsutum* (shoot)	AgNO_3_	–	Shaking 60 °C	20–100	[48]
*Coptis chinensis* (leaf)	AgNO_3_	–	Room temp.; dark 6–45	Room temp.; dark 6–45	[49]

**Table 4:** Silver nanoparticles (AgNPs)–polyacrylamide hydrogel.

Preparation of AgNPs	Size by (nm) for AgNPs	Used Polymer	Method of Incorporation	Summary
**Green-Synthesized chitosan**	240–970	Polyacrylamide (chitosan)	The chitosan solution was mixed with the silver nanoparticles	Hydrogel (S/C-SNPs G-1) demonstrated bactericidal activity [54]

**Table 5:** Frequency of bacteria tested for wound treatment.

Cell Species	AA *	RA (%) *	References
***S. aureus***	24	85.7	[1,56,57]
***E. coli***	22	78.6	[1,56,57,58]

* AA—absolute abundance, RA—relative abundance.

## References

[1] Augustine R., Kalarikkal A., Thomas S. (2016). PCL membranes incorporated with biosynthesized silver nanoparticles as antibacterial wound dressings. Appl. Nanosci..

[2] García-Villén F., Faccendini A., Aguzzi C., Cerezo P., Bonferoni M.C., Rossi S., Grisoli P., Ruggeri M., Ferrari F., Sandri G. (2019). Montmorillonite-Norfloxacin nanocomposite intended for healing of infected wounds. Int. J. Nanomed..

[3] Pang S., Gao Y., Wang F., Wang Y., Cao M., Zhang W., Liang Y., Song M., Jiang G. (2020). Toxicity of silver nanoparticles on wound healing: A case study of zebrafish fin regeneration model. Sci. Total Environ..

[4] Satarupa B., Kumari V., Shatabdi D., Moumita., Dutta D.M., Jyotsna M., Sandhimita M., Arnab G. (2020). Antibacterial, anti-biofilm activity and mechanism of action of pancreatin doped zinc oxide nanoparticles against methicillin resistant *Staphylococcus aureus*. Colloid Surf. B Biointerfaces.

[5] Kardan-Yamchi J., Kazemian H., Battaglia S., Abtahi H., Foroushani A.R., Hamzelou G., Cirillo D.M., Ghodousi A., Feizabadi M.M. (2020). Whole genome sequencing results associated with minimum inhibitory concentrations of 14 anti-tuberculosis drugs among rifampicin-resistant isolates of mycobacterium tuberculosis from Iran. J. Clin. Med..

[6] Natan M., Banin E.N. (2017). From Nano to Micro: Using nanotechnology to combat microorganisms and their multidrug resistance. FEMS Microbiol. Rev..

[7] Shehabeldine A.M., Ashour R.M., Okba M.M., Saber F.R. (2020). Callistemon citrinus bioactive metabolites as new inhibitors of methicillin-resistant *Staphylococcus aureus* biofilm formation. J. Ethnopharmacol..

[8] Halawani E.M., Hassan A.M., Gad E.-R.S. (2020). Nanoformulation of biogenic cefotaxime-conjugated-silver nanoparticles for enhanced antibacterial efficacy against multidrug-resistant bacteria and anticancer studies. Int. J. Nanomed..

[9] Hamida R.S., Abdelmeguid N.E., Ali M.A., Bin-Meferij M.M., Khalil M.I. (2020). Synthesis of silver nanoparticles using a novel cyanobacteria desertifilum sp. extract: Their antibacterial and cytotoxicity effects. Int. J. Nanomed..

[10] Liao C., Li Y., Tjong S.C. (2019). Bactericidal and cytotoxic properties of silver nanoparticles. Int. J. Mol. Sci..

[11] Mounyr B., Moulay S., Saad K.I. (2016). Methods for *in vitro* evaluating antimicrobial activity: A review. J. Pharm. Anal..

[12] Schumacher A., Vranken T., Malhotra A., Arts J.J.C., Habibovic P. (2018). In Vitro antimicrobial susceptibility testing methods: Agar dilution to 3D tissue-engineered models. Eur. J. Clin. Microbiol. Infect. Dis..

[13] Mustapha T., Misni N., Ithnin N.R., Daskum A.M., Unyah N.Z. (2022). A review on plants and microorganisms mediated synthesis of silver nanoparticles, role of plants metabolites and applications. Int. J. Environ. Res. Public Health.

[14] Mandeep K., Ayushi G., Praveen G., Kulvinder S., Vineet K. (2022). Green synthesis of metal nanoparticles and their environmental applications. Curr. Opin. Environ. Sci. Health.

[15] Jaison J., Siaw F.K., Stephen B.-A., Sie Y.L., Ahmed B., Michael K., Danquah J.R. (2022). Green approaches for the synthesis of metal and metal oxide nanoparticles using microbial and plant extracts. Nanoscale.

[16] El Shafey A.M. (2020). Green synthesis of metal and metal oxide nanoparticles from plant leaf extracts and their applications: A review. Green Process Synth..

[17] Sunday A.A., Aderonke S.F., Olabisi T.A. (2019). Instrumental characterization and antibacterial investigation of silver nanoparticles synthesized from *Garcinia kola* leaf. J. Drug Deliv. Ther..

[18] Lee N.-Y., Ko W.-C., Hsueh P.-R. (2019). Nanoparticles in the treatment of infections caused by multidrug-resistant organisms. Front. Pharmacol..

[19] Rodrigo C., Maria B. (2011). Do age and professional rank influence the order of authorship in scientific publications? Some evidence from a micro-level perspective. Scientometrics.

[20] Harris C., Alison C.K. (2012). The overview of reviews: Unique challenges and opportunities when research syntheses are the principal elements of new integrative scholarship. Am. Psychol..

[21] Saeid T.F., Ali R., Sajjad M. (2017). A novel green synthesis of nickel oxide nanoparticles using Arabic gum. Chem. J. Mol..

[22] Raktima C., Sarkar S., Amit K.D., Yuksel A., Saumya D., Madhumita M. (2022). Scope and challenges for green synthesis of functional nanoparticles. Novel Applications of Carbon Based Nano-Materials.

[23] Saravanan M., Arokiyaraj S., Lakshmi T., Pugazhendhi A. (2018). Saravanan. Synthesis of silver nanoparticles from phenerochaete chrysosporium (MTCC-787) and their antibacterial activity against human pathogenic bacteria. Microb. Pathog..

[24] Sehnal K., Hosnedlova B., Docekalova M., Stankova M., Uhlirova D., Tothova Z., Kepinska M., Milnerowicz H., Fernandez C., Ruttkay-Nedecky B. (2019). An assessment of the effect of green synthesized silver nanoparticles using sage leaves (*Salvia officinalis* L.) on germinated plants of maize (*Zea mays* L.). Nanomaterials.

[25] Amr E.-W., Ghada S., Abd E.-G., Sabah A., Abo E., Mervet G.H. (2022). Cytotoxicity and promising anti-biofilm of Curcuma silver nanoparticles against Candida albicans. Res. J. Pharm. Technol..

[26] Oves M.M.O., Mohd A.R., Mohammad A., Huda A.Q., Hana S., Irfan A., Gaffar S.Z., Mohd S. (2022). Green synthesis of silver nanoparticles by Conocarpus lancifolius plant extract and their antimicrobial and anticancer activities. Saudi. J. Biol. Sci..

[27] Eva S.-H., Vicente G.-G., Adriana C.-G., José C.-G., Jesús M.-G., Pablo M.-R. (2023). Phytochemical profle and activity against Fusarium species of Tamarix gallica bark aqueous ammonia extract. Agronomy.

[28] Chinnappan S., Kandasamy S., Arumugam S., Seralathan K.K., Thangaswamy S., Muthusamy G. (2018). Biomimetic synthesis of silver nanoparticles using fower extract of Bauhinia purpurea and its antibacterial activity against clinical pathogens. Env. Sci. Poll. Res..

[29] Nelson D., Priscyla D., Marcato G.I.H.S., Oswaldo L.A. (2007). Antibacterial Effect of Silver Nanoparticles Produced by Fungal Process on Textile Fabrics and Their Effluent Treatment. J. Biomed. Nanotechnol..

[30] Sundaramoorthi C., Dhivya M.M., Palanisamy S., Vivekanandan K. (2009). Biosynthesis of silver nanoparticles from Aspergillus niger and evaluation of its wound healing activity in experimental rat model. Int. J. PharmTech Res..

[31] Wankar S., Walake S., Gumathannavar R., Sapre N., Kulkarni A. (2022). The era of green nanomaterials for sensing. Innovations in Green Nanoscience and Nanotechnology.

[32] Dat N.M., Thinh D.B., Huong L.M., Tinh N.T., Linh N.T.T., Hai N.D., Viet N.D., Dat N.T., Phong M.T., Hieu N.H. (2022). Facile synthesis and antibacterial activity of silver nanoparticles-modifed graphene oxide hybrid material: The assessment, utilization, and anti-virus potentiality. Mat. Today Chem..

[33] Moldovan B., David L., Vulcu A., Olenic L., Perde-Schrepler M., Fischer-Fodor E., Baldea I., Clichici S., Filip G.A. (2017). In vitro and in vivo anti-infammatory properties of green synthesized silver nanoparticles using *Viburnum opulus* L. fruits extract. Mat. Sci. Eng. C.

[34] Tanja K., Joerger R., Olsson E., Claes-Göran G. (1999). Silver-based crystalline nanoparticles, microbially fabricated. Proc. Natl. Acad. Sci. India Sect. B Biol. Sci..

[35] Deshpande L.M., Chopade B.A. (1994). Plasmid mediated silver resistance in Acinetobacter baumannii. Biometals.

[36] Ali J., Ali N., Wang L., Waseem H., Pan G. (2019). Revisiting the mechanistic pathways for bacterial mediated synthesis of noble metal nanoparticles. J. Microbiol. Methods.

[37] Galvez A.M., Ramos K.M., Alexis J.T., Baculi R. (2019). Bacterial exopolysaccharide-mediated synthesis of silver nanoparticles and their application on bacterial biofilms. J. Microbiol. Biotechnol. Food Sci..

[38] Hina S., Juan D., Priyanka S., Tae H.Y. (2018). Extracellular synthesis of silver nanoparticles by *Pseudomonas* sp. THG-LS1. 4 and their antimicrobial application. J. Pharm. Anal..

[39] Palaniappan S., Palaniappan S., Subramaniam P., Murugesan S., Tamilselvi M., Loganathan S., Kannan S., Thangavel B. (2018). Characterization, antimicrobial and antioxidant property of exopolysaccharide mediated silver nanoparticles synthesized by Streptomyces violaceus MM72. Carbohydr. Polym..

[40] Ahmed A.A., Hamzah H., Maaroof M. (2018). Analyzing formation of silver nanoparticles from the filamentous fungus Fusarium oxysporum and their antimicrobial activity. Turk. J. Biol..

[41] Shivani T.J., Gade A.C. (2015). Biosynthesis of silver nanoparticles using *Bacillus* sp. for Microbial Disease Control: An in-vitro and in-silico approach. Sch. Acad. J. Pharm..

[42] Anima N., Saef M., Mohammed T.A., Gouri K.D. (2015). Fungal mediated synthesis of silver nanoparticles andits role in enhancing the bactericidal property of Amoxicillin. Der. Pharm. Lett..

[43] Salaheldin T.A., Husseiny S.M., Al-Enizi A.M., Elzatahry A., Cowley A.H. (2016). Evaluation of the cytotoxic behavior of fungal extracellular synthesized Ag nanoparticles using confocal laser scanning microscope. Int. J. Mol. Sci..

[44] Neethu S., Midhun S.J., Radhakrishnan E.K., Jyothis M. (2018). Green synthesized silver nanoparticles by marine endophytic fungus Penicillium polonicum and its antibacterial efficacy against biofilm forming, multidrug-resistant Acinetobacter baumanii. Microb. Pathog..

[45] Vahideh A., Iman S., Morteza Y., Zahra G. (2018). Mangrove-mediated synthesis of silver nanoparticles using native Avicennia marina plant extract from southern Iran. Chem. Eng. Commun..

[46] Tippayawat P., Phromviyo N., Boueroy P., Chompoosor A. (2016). Green synthesis of silver nanoparticles in aloe vera plant extract prepared by a hydrothermal method and their synergistic antibacterial activity. PeerJ.

[47] Siddhant J., Mohan S.M. (2017). Medicinal plant leaf extract and pure flavonoid mediated green synthesis of silver nanoparticles and their enhanced antibacterial property. Sci. Rep..

[48] Gulamnabi L., Vanti V.B., Nargund B.K.N., Rajinikanth V., Mahantesh K., Sikandar I.M., Suresh T., Rajashekar R.P. (2019). Synthesis of Gossypium hirsutum-derived silver nanoparticles and their antibacterial efficacy against plant pathogens. Appl. Organomet. Chem..

[49] Dziedzic A., Kubina R., Bułdak R.J., Skonieczna M., Cholewa K. (2016). Silver nanoparticles exhibit the dose-dependent anti-proliferative effect against human squamous carcinoma cells attenuated in the presence of berberine. Molecules.

[50] Haleem A., Chen J., Guo X.-X., Wang J.-Y., Li H.-J., Li P.-Y., Chen S.-Q., He W.-D. (2020). Hybrid cryogels composed of P(NIPAM-co-AMPS) and metal nanoparticles for rapid reduction of p-nitrophenol. Polymer.

[51] Aldakheel F.M., Mohsen D., El Sayed M.M., Alawam K.A., Binshaya A.S., Alduraywish S.A. (2023). Silver Nanoparticles Loaded on Chitosan-g-PVA Hydrogel for the Wound-Healing Applications. Molecules.

[52] Saberi A., Mohammad S., Eskandar A. (2020). Design of AgNPs-Base Starch/PEG-Poly (Acrylic Acid) Hydrogel for Removal of Mercury (II). J. Polym. Environ..

[53] Narjes N.D., Mohammad S. (2018). Free radical synthesis of nanosilver/gelatin-poly (acrylic acid) nanocomposite hydrogels employed for antibacterial activity and removal of Cu (II) metal ions. J. Hazard. Mater..

[54] Verma J., Kanoujia J., Parashar P., Tripathi C.B., Saraf S.A. (2017). Wound healing applications of sericin/chitosan-capped silver nanoparticles incorporated hydrogel. Drug Deliv. Translat. Res..

[55] Vichayarat R., Nuttaporn P., Pitt S. (2012). In Vitro efficacy and toxicology evaluation of silver nanoparticle-loaded gelatin hydrogel pads as antibacterial wound dressings. J. Appl. Polymer. Sci..

[56] Sudheesh Kumar P.T., Abhilash S., Manzoor K., Nair S.V., Tamura H., Jayakumar R. (2010). Preparation and characterization of novel β-chitin/nanosilver composite scaffolds for wound dressing applications. Carbohydr. Polym..

[57] Das A., Kumar A., Patil N.B., Viswanathan C., Ghosh D. (2015). Preparation and characterization of silver nanoparticle loaded amorphous hydrogel of carboxymethylcellulose for infected wounds. Carbohydr. Polym..

[58] Varaprasad K., Varaprasad Y., Murali M.S., Ravindra N., Narayana R., Vimala K., Monika K., Sreedhar B., Mohana R.K. (2010). Hydrogel–Silver nanoparticle composites: A new generation of antimicrobials. J. Appl. Polymer Sci..

[59] Zhang D., Ma X.L., Gu Y., Huang H., Zhang G.W. (2020). Green synthesis of metallic nanoparticles and their potential applications to treat cancer. Front. Chem..

[60] Arpita R., Chetan P., Amel G., Mohammed S., Alqahtani M.B., Saiful I., Jamal H.M., Mohammed J. (2022). Biologically derived gold nanoparticles and their applications. Bioinorg. Chem. Appl..

[61] AMosquera-Romero S., Anaya-Garzon J., Garcia-Timermans C., Van D.J., Hoorens A., Commenges-Bernole N., Verbeken K., Rabaey K., Varia J. (2022). Combined gold recovery and nanoparticle synthesis in microbial systems using fractional factorial design. Nanomaterials.

[62] Karade V.C., Dongale T.D., Subasa C.S., Kollu P., Chougale A.D., Patil P.S., Patil P. (2018). B. Effect of reaction time on structural and magnetic properties of green-synthesized magnetic nanoparticles. J. Phy. Chem. Sol..

[63] Razali Z., Norrizah J.S., Abdullah S. (2022). Impact of temperature and pH on antioxidant activity of green silver nanoparticles fabricated from Ananas comosus peel extracts. IOP Conf. Ser. Earth Environ. Sci..

[64] Habeeb R.H., Dhandapani R., Narayanan S., Palanivel V., Paramasivam R., Subbarayalu R., Thangavelu S., Muthupandian S. (2022). Medicinal plants mediated the green synthesis of silver nanoparticles and their biomedical applications. IET Nanobiotechnol..

[65] Mohd Q.K., Praveen K., Rais A.K., Khursheed A. (2022). Fabrication of sulfur-doped reduced graphene oxide modifed glassy carbon electrode (S@ rGO/GCE) based acetaminophen sensor. Inorganics.

[66] Liyana R., Kamalrul A.A., Rajinder S., Sharifah N.S.J., Abrizah O., Wolfram W., Umi S.R. (2023). Fourier transform infrared (FTIR) spectroscopy approach combined with discriminant analysis and prediction model for crude palm oil authentication of diferent geographical and temporal origins. Food Control..

[67] Naganthran A., Verasoundarapandian G., Khalid F.E., Masarudin M.J., Zulkharnain A., Nawawi N.M., Karim M., Che Abdullah C.A., Ahmad S.A. (2022). Synthesis, characterization and biomedical application of silver nanoparticles. Materials.

[68] Nahari M.H., Al A.A., Asiri A., Mahnashi M.H., Shaikh I.A., Shettar A.K., Hoskeri J. (2022). Green synthesis and characterization of iron nanoparticles synthesized from aqueous leaf extract of Vitex leucoxylon and its biomedical applications. Nanomaterials.

[69] Wu X., Fang F., Zhang B., Wu J., Zhang K. (2022). Biogenic silver nanoparticles-modifed forward osmosis membranes with mitigated internal concentration polarization and enhanced antibacterial properties. NPJ Clean Water.

[70] Nisreen J.A., Ekhlas A.A.A.G., Nuha A.I. (2023). Eco-friendly approach for silver nanoparticles synthesis from lemon extract and their anti-oxidant, anti-bacterial, and anti-cancer activities. J. Turk. Chem. Soc. Sect A Chem..

[71] Leila C., Sanjeev N., Julie M., Wendy H., Karina D., Roy M.K. (2007). A retrospective cohort study of ActicoatTM versus SilvazineTM in a paediatric population. Burns.

[72] Jones S.A., Bowler P.G., Walker M., Parsons D. (2004). Controlling wound bioburden with a novel silver-containing HydrofiberR dressing. Wound Repair Regen..

[73] Hira C., Manisha P., Yan Q.L., Chea Y.L., Cheng T.L., Tee C.L.M., Huai S.L., Yee P.L., Cheng F.L., Subrat K.B. (2020). Silver nanoparticles: Advanced and promising technology in diabetic wound therapy. Mater. Sci. Eng. C.

[74] Meekul J., Chotirosniramit A., Himakalasa W., Orrapin S., Wongthanee A., Pongtam O., Kulprachakarn K., Rerkasem K. (2017). A Randomized Controlled Trial on the Outcome in Comparing an Alginate Silver Dressing with a Conventional Treatment of a Necrotizing Fasciitis Wound. Int. J. Low. Extrem. Wounds.

[75] Hahn H.M., Lee I.J., Woo K.J., Park B.Y. (2019). Silver-Impregnated Negative-Pressure Wound Therapy for the Treatment of Lower-Extremity Open Wounds: A Prospective Randomized Clinical Study. Adv. Ski. Wound Care.

[76] Chen J., Zou Q., Hamblin M.R., Wen X. (2021). A preliminary clinical trial comparing wet silver dressings versus wet-to-dry povidone-iodine dressings for wound healing in pemphigus vulgaris patients. Dermatol. Ther..

[77] Karlsson M., Elmasry M., Steinvall I., Sjöberg F., Olofsson P., Thorfinn J. (2019). Superiority of silver-foam over porcine xenograft dressings for treatment of scalds in children: A prospective randomised controlled trial. Burns.

[78] Akin T., Kendirci M., Akgün A.E., Cetinkaya E., Er S., Akin M., Yasti A.C. (2022). Applying a Silver-containing Dressing to the Incision Site and Its Effect on the Development of Surgical Site Infection After Ostomy Closure: A Prospective Randomized Clinical Pilot Study. Wound Manag. Prev..

[79] Gopinath P., Gogoi S.K., Chattopadhyay A., Ghosh S.S. (2008). Gopinath. Implications of silver nanoparticle induced cell apoptosis for in vitro gene therapy. Nanotechnology.

[80] Borm P.J., Kreyling W. (2004). Toxicological hazards of inhaled nanoparticles—Potential implications for drug delivery. J. Nanosci. Nanotechnol..

[81] Megan E.M., Melissa J.P. (2007). Are nanoparticles potential male reproductive toxicants? A literature review. Nanotoxicology.

[82] Hussain S.M., Javorina A.K., Schrand A.M., Duhart H.M., Ali S.F., Schlager J.J. (2006). The interaction of manganese nanoparticles with PC-12 cells induces dopamine depletion. Toxicol. Sci..

[83] Hussain S.M., Hess K.L., Gearhart J.M., Geiss K.T., Schlager J. (2005). In Vitro toxicity of nanoparticles in BRL 3A rat liver cells. Toxicol. Vitr..

[84] Braydich-Stolle L., Hussain S., Schlager J.J., Hofmann M.C.B.-S. (2005). In Vitro cytotoxicity of nanoparticles in mammalian germline stem cells. Toxicol. Sci..

[85] Chen K., Wang F., Liu S., Wu X., Xu L., Zhang D. (2020). In Situ reduction of silver nanoparticles by sodium alginate to obtain silver-loaded composite wound dressing with enhanced mechanical and antimicrobial property. Int. J. Biol. Macromol..

[86] Ip M., Lui S.L., Poon V.K.M., Lung I., Burd A. (2006). Antimicrobial activities of silver dressings: An in vitro comparison. J. Med. Microbiol..

[87] Sougata S., Atish D.J., Samir K.S., Golam M. (2007). Facile synthesis of silver nano particles with highly efficient anti-microbial property. Polyhedron.

[88] Kokura S., Handa O., Takagi T., Ishikawa T., Naito Y., Yoshikawa T. (2010). Silver nanoparticles as a safe preservative for use in cosmetics. Nanomed. Nanotechnol. Biol. Med..

[89] Yang Y., Qin Z., Zeng W., Yang T., Cao Y., Mei C., Kuang Y. (2017). Toxicity assessment of nanoparticles in various systems and organs. Nanotechnol. Rev..

[90] Berhanu W.Z. (2016). A review of stabilized silver nanoparticles–synthesis, biological properties, characterization, and potential areas of applications. Nanomed.

[91] Rai M.K., Deshmukh S.D., Ingle A.P., Gade A.K. (2012). Silver nanoparticles: The powerful nanoweapon against multidrug-resistant bacteria. J. Appl. Microbiol..

[92] Atiyeh B.S., Costagliola M., Hayek S.N., Dibo S.A. (2007). Effect of silver on burn wound infection control and healing: Review of the literature. Burns. Burns.

[93] Franková J., Pivodová V., Vágnerová H., Juráňová J., Ulrichová J. (2016). Effects of silver nanoparticles on primary cell cultures of fibroblasts and keratinocytes in a wound-healing model. J. Appl. Biomater. Funct. Mater..

[94] Rajalekshmi R., Sivan U., Lissy K.K., Kalliyana K.V. (2017). Synthesis and characterization of silver nanoparticle incorporated gelatin-hydroxypropyl methacrylate hydrogels for wound dressing applications. J. Appl. Polym. Sci..

[95] Katayoon K., Ebrahim M., Thomas J.W., Amalina B.M.A. (2020). Wound dressings functionalized with silver nanoparticles: Promises and pitfalls. Nanoscale.

[96] Ágnes S., Margarita P., Krassimira Y., Judit M., Judith M., Pavletta S. (2014). Silver-and sulfadiazine-loaded nanostructured silica materials as potential replacement of silver sulfadiazine. J. Mater. Chem. B.

[97] Walker M., Cochrane C.A., Bowler P.G., Parsons D., Bradshaw P. (2006). Silver deposition and tissue staining associated with wound dressings containing silver. Ostomy Wound Manag..

[98] Trop M., Novak M., Rodl S., Hellbom B., Kroell W., Goessler W. (2006). Silver-Coated dressing acticoat caused raised liver enzymes and argyria-like symptoms in burn patient. J. Trauma Acute Care Surg..

[99] Anisha B.S., Biswas R., Chennazhi K.P., Jayakumar R. (2013). Chitosan–Hyaluronic acid/nano silver composite sponges for drug resistant bacteria infected diabetic wounds. Int. J. Biol. Macromol..

[100] Geewoo N., Sabarinathan R., Baskaran P., Joon M.S. (2015). The application of bactericidal silver nanoparticles in wound treatment. Nanomater. Nanotechnol..

[101] Zhang Z., Zhang M., Chen S., Horbett T.A., Ratner B.D., Jiang S. (2008). Blood compatibility of surfaces with superlow protein adsorption. Biomaterials.

[102] Cai N., Li Q., Zhang J., Xu T., Zhao W., Yang J., Zhang L. (2017). Antifouling zwitterionic hydrogel coating improves hemocompatibility of activated carbon hemoadsorbent. J. Colloid Interface Sci..

[103] Wensheng C., Eric C.A., Ram B.G. (2001). Separation of lignin from aqueous mixtures by ionic and nonionic temperature-sensitive hydrogels. Ind. Eng. Chem. Res..

[104] Zhang W., Sun Y., Zhang L. (2015). In situ synthesis of monodisperse silver nanoparticles on sulfhydryl-functionalized poly(glycidyl methacrylate) micro-spheres for catalytic reduction of 4-nitrophenol. Ind. Eng. Chem. Res..

[105] Murali M.Y., Kyungjae L., Thathan P., Kurt E.G. (2007). Hydrogel networks as nanoreactors: A novel approach to silver nanoparticles for antibacterial applica-tions. Polymer.

[106] Zhang P., Shao C., Zhang Z., Zhang M., Mu J., Guo Z., Liua Y. (2011). “In situ assembly of well-dispersed Ag nanoparticles (AgNPs) on electrospun carbon nanofibers (CNFs) for catalytic re-duction of 4-nitrophenol. Nanoscale.

[107] Song J., Zhu Y., Zhang J., Yang J., Du Y., Zheng W., Wen C., Zhang Y., Zhang L. (2019). Encapsulation of AgNPs within Zwitterionic hydrogels for highly efficient and antifouling catalysis in biological environ-ments. Langmuir.

[108] Shao J., Wang B., Li J., John A.J., Walboomers X.F., Yang F. (2019). Antibacterial effect and wound healing ability of silver nanoparticles incorporation into chitosan-based nanofibrous mem-branes. Mater. Sci. Eng. C Mater. Biol. Appl..

